# Meta-narrative and realist reviews: guidance, rules, publication standards and quality appraisal

**DOI:** 10.1186/1741-7015-11-22

**Published:** 2013-01-29

**Authors:** David Gough

**Affiliations:** 1 EPPI-Centre, Social Science Research Unit, Institute of Education, University of London, 18 Woburn Square, London, WC1H 0NR, UK

**Keywords:** Systematic reviews, Meta-narrative, Realist synthesis.

## Abstract

Recently, there has been an expansion of different forms of systematic review of research and the development of guidance and standards about particular types of review. These reviews can be best understood within a broad framework of the dimensions on which reviews differ, and how the review methodology relates to the methodology of primary research. Similarly, publication standards can be understood in terms of their relation to other standards such as guidance and rules for undertaking reviews and systems for appraising the quality of reviews. This commentary is written with special reference to the publication standards for meta-narrative and realist reviews being published in *BMC Medicine*.

See related research articles http://www.biomedcentral.com/1741-7015/11/20 and http://www.biomedcentral.com/1741-7015/11/21

## Commentary

### Expansion in methods of systematic review

Primary research and reviews of such research both address similar research questions and use similar methodological approaches. Methodological debates in relation to reviews of research tend to reflect similar debates in primary research. In many cases, research reviews employ methodologies similar to the methodologies of the studies that they review. So, for example, statistical meta analysis of randomized controlled trials has similarities with the analysis of individual trials. Also, meta ethnography uses methods of analysis that relate to ethnography.

In the health and social sciences there is a rich diversity of these research questions and methodologies. Until recently, however, the term systematic review has been seen by some as synonymous with reviews assessing the efficacy of interventions by considering experimentally controlled outcome studies. The burgeoning of different methods of review is therefore to be welcomed [1,2]. Reviews now vary in their questions and theoretical and ideological assumptions, general methodological approach, specific methods, studies included, the components of the review, and the breadth and depth and 'work done' by the review [3,4]. The extent of this possible variation suggests the need for a specification of the dimensions of difference between reviews rather than a simple classification of review types.

One important dimension of difference is whether reviews ask questions and use methodologies that predominantly aim to aggregate information with a prior determined conceptual framework, or whether they predominantly interpret, configure and arrange theories and concepts. Aggregative reviews tend to use *a priori *methods using predefined concepts and their findings are used to inform instrumental decision making; configuring reviews tend to use iterative methods using emergent concepts and their findings are used to inform through enlightenment [3-5]. This distinction between aggregating and configuring has some similarities to, but does not map directly on to, the rather imprecise distinction between quantitative and qualitative research [5].

This commentary considers two specific types of systematic review, meta-narrative synthesis and realist synthesis, to accompany the publication in *BMC Medicine *of publication standards for the reporting of such reviews. Both of these approaches ask broad questions and consider many types of evidence to address them and so for these reviews their methodologies do not necessarily reflect the methodologies of the primary studies. The breadth of the review questions addressed in these reviews also creates challenges for the reporting the depth of detail in each part of the review to provide the necessary transparency for accountability and quality appraisal.

### Meta-narrative reviews

Meta-narrative reviews 'look historically at how particular research traditions have unfolded over time and shaped the kind of questions being asked and the methods used to answer them' [6,7]. They examine the range of approaches to studying an issue, interpret and create an account of the development of these separate 'meta-narratives' and then create an overarching meta-narrative summary. The configuration of different research traditions on the same topic is also a feature of some other approaches such as meta triangulation [8] and meta-theoretical reviews [9].

Using the language of dimensions of difference, meta-narrative reviews can be described as having three overlapping levels (or components). First is an iterative configuring map of the different traditions of research in an area. Second is an iterative configuring and/or aggregative analysis (synthesis) of different traditions within the map. Third is an iterative configuring comparison and contrast of the different meta-narratives to create an overarching synthesis of the whole map.

The approach is predominantly configuring (rather than aggregative) and iterative (rather than *a priori *in method). Searching, for example, is an iterative exploration of studies, ideas and data that inform the development of the meta-narrative stories rather than an exhaustive search for all studies from a research tradition. The scope of the whole review and each of the three components (or levels of analysis) can vary as the work progresses.

The reviews are primarily concerned with how issues were researched rather than synthesizing the findings and so can be considered a form of multi-level configuring mapping rather than synthesis of research findings. To the extent that some meta-narrative reviews may consider study findings, then this analysis could involve an interpretative configuring of ideas and the aggregation of data.

### Realist reviews

Realist reviews are used to evaluate the mechanisms, contexts and outcomes of middle range theories and social policies [10,11]. They thus have a broader focus than review questions narrowly addressing the efficacy of specific interventions and particularly of theory free ('black box') review questions. The approach is sometimes described as 'what works for whom under what circumstances and why,'; though this is an ambition also shared by other theory-driven approaches to the evaluation of complex interventions in which all or part of a logic model is examined to test if and why an intervention might have certain effects [12,13]. Many other theory-driven approaches to evaluation are also realist in the philosophical sense in that they assume that an external reality exists even if this cannot be directly known [2].

Realist reviews are different from many other theory-driven reviews in using an exploratory iterative approach to examining the links between context, mechanism and outcome (CMO) in a similar way to realist evaluation in primary research [14]. Realist reviews also unpack the causal model as part of the review process rather than as a prior developmental stage. They then test parts of the model using an iterative investigative stance rather than the more *a priori *approach of standard empirical testing. Another review method, critical interpretive synthesis also has these characteristics of combining configuring theory and iterative investigation with aggregative analysis [15].

Using the language of dimensions of difference, realist reviews can be described as iterative multi-component mixed method reviews (configuring and aggregative) with three overlapping main components. First is an iterative configuring process of unpacking the explicit and implicit assumptions of context, mechanism and outcome for a particular mid-level theory. Second is an iterative aggregative testing of the empirical data on particular CMO configurations. Third is an iterative configuring across these CMO configurations to explore and explain different findings in different contexts.

### Guidelines, publication standards and quality appraisal for reviews

The publication of publication standards for reviewing raises several issues about how they relate to both guidelines (and rules) for undertaking reviews and to methods for the quality appraisal of reviews.

A first issue concerns how publication standards relate to guidelines in terms of explaining what is required to be undertaken in such a review. The publication standards for meta-narrative and realist reviews focus on what should be reported about the review. They indirectly give many insights into the nature of the methods but a detailed explanation of the rationale for the method and how to undertake the reviews is also required (and is planned for meta-narrative and realist reviews).

A second issue is that guidelines about how to undertake and report reviews can also be rules. There is an obvious concern that reviews should be well executed using fit-for-purpose methods and guidance and standards can inform authors, editors and reviewers. Many organizations also have mechanisms such as methods groups to both develop new methods and manage review quality. This can be achieved through training, statements of expectations (such as the Methodological Expectations of Cochrane Intervention Reviews (MECIR) [16]), feedback and advice and by gate keeping whether the final product is published as a review from that organization.

A third issue is the role of procedures for the quality appraisal of reviews. AMSTAR (A Measurement Tool to Assess Systematic Reviews) [17], for example, is a system for appraising the quality of aggregative reviews of efficacy of interventions. Such appraisal is likely to be based on both the quality and fitness for purpose of the review and the quality (or standard) of the reporting. If quality can be assessed through guidance and rules for undertaking and reporting a review, then maybe the main rationale for a separate quality appraisal system is the assessment of the fitness for purpose of that method for the question being addressed.

A fourth issue is the feedback of these guidance, rules and expectations, publication standards and quality appraisal of reviews on the planning of primary research. Those involved in reviews are concerned about the quality of primary research and may develop guidance on the execution and reporting of research to ensure that it is fit for purpose (for example, the CONSORT (Consolidated Standards of Reporting Trials) statement [18]). Even without such guidance, primary researchers may plan their studies with consideration as to whether they will be included in a review. Furthermore, primary studies are not only designed in terms of what they can contribute on their own but in terms of how much they can contribute to updating and developing and changing the conclusions to an existing systematic review. For example, making power calculation judgments for a randomized controlled trial on the basis of the power that would be required to change the conclusions of a pre-existing statistical meta-analysis.

A fifth issue is that these systems of guidance, rules, reporting standards and appraisal vary in the extent of their coverage of review types and the clarity with which this coverage is labelled and explained. Some are review type specific. Others, such as the PRISMA (Preferred Reporting Items for Systematic Reviews and Meta-analysis) statement on review reporting [19] and AMSTAR on review appraisal systems cover a range of aggregative reviews.

A final overarching issue is the branding of reviews and the distinction between these review types. The move to label different review types can provide essential detail about individual methodology as in the case of meta-narrative and realist reviews. However, there is also the danger that a plethora of named types of review may obscure similar equally useful methods. At a time when review methods are developing quickly, when current approaches are influencing each other, and when review methodology may seem very different in a few years, it is important to maintain a wider conceptualization of review approaches and methods and the dimensions on which they differ. The research questions posed by both meta-narrative and realist reviews can provide some pointers for this. We can consider the development of research traditions of research reviews and how they relate with each other. We can also examine the contexts, mechanisms and outcomes by which these approaches and specific methods achieve their aims. There is a richness of approaches to be used and developed as long as we keep to the core principles of fitness for purpose, rigor in execution and transparency and completeness in reporting.

## Competing interests

DG is active in undertaking reviews and developing review methods. He has no other competing interests.

## Author's information

DG works at the EPPI-Centre which is active in undertaking reviews and developing review methods.

## Pre-publication history

The pre-publication history for this paper can be accessed here:

http://www.biomedcentral.com/1741-7015/11/22/prepub
